# Effects of fungal seed endophyte FXZ2 on *Dysphania ambrosioides* Zn/Cd tolerance and accumulation

**DOI:** 10.3389/fmicb.2022.995830

**Published:** 2022-09-21

**Authors:** Vijay K. Sharma, Shobhika Parmar, Wenting Tang, Haiyan Hu, James F. White, Haiyan Li

**Affiliations:** ^1^Medical School, Kunming University of Science and Technology, Kunming, China; ^2^State Key Laboratory of Environmental Geochemistry, Institute of Geochemistry, Chinese Academy of Sciences, Guiyang, China; ^3^Department of Plant Biology, Rutgers University, New Brunswick, NJ, United States

**Keywords:** seed endophytes, *Dysphania ambrosioides*, metal stress, phytoremediation, mechanism, chemical speciation

## Abstract

Metal-induced oxidative stress in contaminated soils affects plant growth. In the present study, we evaluated the role of seed endophyte FXZ2 on *Dysphania ambrosioides* Zn/Cd tolerance and accumulation. A series of pot experiments were conducted under variable Zn (500, 1,000, and 1,500 mg kg^–1^) and Cd (5, 15, 30, and 60 mg kg^–1^). The results demonstrated that FXZ2-inoculation significantly enhanced the growth of *D. ambrosioides* and improved its chlorophyll and GSH content. In the rhizosphere, FXZ2 inoculation changed the chemical speciation of Zn/Cd and thus affected their uptake and accumulation in host plants. The exchangeable and carbonate-bound fractions (F1 + F2) of Zn decreased in the rhizosphere of FXZ2-inoculated plants (E+) as compared to non-inoculated plants (E-) under Zn stress (500 and 1,000 mg kg^–1^), correspondingly, Zn in the shoots of E+ decreased (*p* < 0.05). However, at Cd stress (30 and 60 mg kg^–1^), the F1 + F2 fractions of Cd in E+ rhizospheric soils increased; subsequently, Cd in the shoots of E+ increased (*p* < 0.05). FXZ2 could exogenously secrete phytohormones IAA, GA, and JA. The study suggests that seed endophyte FXZ2 can increase Zn/Cd tolerance of host plant by altering Zn/Cd speciation in rhizospheric soils, as well as exogenous production of phytohormones to promote growth, lowering oxidative damage while enhancing antioxidant properties. For Zn/Cd accumulation, it has opposite effects: Zn uptake in E+ plants was significantly (*p* < 0.05) decreased, while Cd accumulation in E+ plants was significantly (*p* < 0.05) increased. Thus, FXZ2 has excellent application prospects in Cd phytoextraction and decreasing Zn toxicity in agriculturally important crops.

## Introduction

Plants rely on various metals for normal physiology, but higher or excess metals in the soil not only deteriorate the soil health and change the native microbial community but also adversely affect the physiology and metabolism of plants (Kidd et al., 2012; Chen et al., 2014; Parmar and Singh, 2015; Etesami, 2018). Zinc (Zn) is an essential element for plants, but a higher concentration of Zn in the soil adversely affects plant growth via root growth inhibition, mitotic efficiency, chromosomal aberrations as well as oxidative stress (Jain et al., 2010). Cadmium (Cd) is a non-essential trace element that can cause toxicity even at lower concentrations (Wagner, 1993; Nan et al., 2002; Kuriakose and Prasad, 2008), accumulates readily in the soil and enters the food chain via enrichment in food crops (Wang et al., 2022). The bioavailability, mobility, and toxicity of these metals to plants depend on their chemical forms rather than the total contents (Liu et al., 2007). Therefore, the chemical speciation of metals in the soil may have an important impact on plants (Tüzen, 2003; Ahlf et al., 2009).

It is well known that metal-contaminated soils cause various problems to the surrounding environments, such as plants survival, agricultural production, food safety, and human health; therefore, the remediation of these metal-contaminated soils is of utmost importance (Hussain et al., 2022). Some plants growing in highly metal-contaminated environments evolved to tolerate metal stress; they have potential applications in phytoremediation. Previous studies have demonstrated that plant-associated microbes, i.e., endophytes can increase host plants’ metal tolerance properties, enhance their growth, and influence their metal accumulation (Sharma et al., 2019; Rattanapolsan et al., 2021; Ważny et al., 2021; Hussain et al., 2022). It is believed that endophytes induced tolerance and growth improvement of host plants to metal stress by detoxification through chelation and compartmentalization of metal ions, increasing nutrient absorption and root growth, changing the distribution of metal in plant cells, modulating the antioxidative system, and secretion of phytohormones (Bilal et al., 2018; White et al., 2019; Chang et al., 2021; Akhtar et al., 2022).

FXZ2 is a fungal seed endophyte that has been isolated from *Arabis alpina*, and it has been identified to be *Epicoccum nigrum* (GenBank accession number is ON209455) (Chu et al., 2017). Our previous studies have demonstrated that FXZ2 has high tolerance and adsorption capacity for lead (Pb) and Cd, and it can significantly enhance host plants’ growth under Zn/Cd stress. Seed endophytes are attributed to providing beneficial traits such as improving nutrient uptake, reducing susceptibility to drought and temperature stress, and improving the growth of host plants. However, the role of seed endophytes on the plants’ metal tolerance and accumulation as well as its mechanisms are still unknown. For the beneficial characteristics that the seed endophyte can be transferred to the next generation through vertical transmission (Li et al., 2019), therefore, in practice, it has more advantages than the other symbiotic microbes. For example, the seed endophyte RE3-3 *Herbaspirillum frisingense* was successfully transmitted to the next generation seeds of *Phragmites australis* and, consequently, enhanced seedling development and growth under Cd stress (Gao and Shi, 2018).

*Dysphania ambrosioides* (L.) Mosyakin and Clemants is a dominant plant in Pb-Zn mining sites of Huize County, Yunnan Province, China. It has been reported as a Cd-accumulator and a Pb-hyperaccumulator, which showed potential application in phytoremediation of multi-metal-contaminated sites (Wu et al., 2004; Li et al., 2012; Li X. et al., 2016). The present study aimed to investigate the role of fungal seed endophyte FXZ2 on *D. ambrosioides* Zn/Cd tolerance under variable Zn (500, 1,000, and 1,500 mg kg^–1^ soil) and Cd (5, 15, 30, and 60 mg kg^–1^ soil) stress. Further, the speciation of Zn/Cd in rhizospheric soils of *D. ambrosioides* was tested by Tessier sequential extraction methods. The objective of this study is to elucidate how the seed endophyte FXZ2 altered the metals’ chemical speciation in rhizospheric soils and thus affected their absorption, translocation, and accumulation in host plants. The novelty of this work is that it gives important information about the function of seed endophytes in increasing the survival and growth of host plants under metal stress conditions.

## Materials and methods

### Fungal seed endophyte FXZ2

The fungal seed endophyte FXZ2 was previously isolated from the seeds of *Arabis alpina*, which were collected from the Pb-Zn mining sites of Huize County, Yunnan Province, Southwest China (25°28′17″ N, 103°37′34″ E) (Chu et al., 2017). FXZ2 was identified to be *Epicoccum nigrum* based on its morphological features and molecular analysis (Chu et al., 2017), and its GenBank database accession number is ON209455^1^. The isolate showed better Pb and Cd tolerance and adsorption capacity, and has been authorized by the Patent Office of the People’s Republic of China (ZL 2017 1 0028569. 2). It was submitted to the Chinese General Microbiological Culture Collection Center (CGMCC NO.13573).

### Phytohormone production

To assess for phytohormones jasmonic acid (JA), indole-3-acetic acid (IAA), and gibberellic acid (GA) production, the isolate FXZ2 was grown in PDB (potato dextrose broth) at 28 ± 2°C for 21 days in a shaker. After that, the culture was filtered and the broth was collected and extracted three times with ethyl acetate, followed by concentration using a vacuum rotary evaporator. Finally, the extract was dissolved in methanol for phytohormone tests according to the manufacturer of plant hormone kits (MLBIO Biotechnology Co., Ltd., Shanghai). A change in the color of the reaction mixture was measured by a spectrophotometer at a wavelength of 450 nm. And the concentrations of IAA, GA, and JA in the extracts were calculated by comparing the OD of the extracts to the standard curve of the IAA, GA, and JA. Three replicates were performed.

### Pot experiments

The mature seeds were collected from naturally growing *D. ambrosioides* and surface sterilized as Li et al. (2012). Subsequently, the seeds were germinated on a plastic tray that contained a fixed soil substrate (perlite: peat moss, 3:7, vol:vol) in a light incubator (25 ± 1/18 ± 1°C, 16/8 h day/night cycle, 60% relative humidity). Twenty-one days later, the germinated seedlings with equal size were transplanted to the pots (1 seedling/pot), which contained 150 gm of sterilized soil substrate mixed with the overages of ZnSo_4_.7H_2_O or CdCl_2_.2.5H_2_O to the final concentration of 0, 500, 1,000, and 1,500 mg Zn kg^–1^ and 0, 5, 15, and 30 mg Cd kg^–1^, respectively. The pots were kept in a random configuration and exposed to artificial plant lighting (16/8 h day/night cycle). Every 2–3 days, the plants were irrigated with autoclaved water, and once a week Peter’s General Purpose 20-20-20 fertilizer (Grace Sierra Horticultural Products, Milpitas, CA, USA) was given.

For the inoculation, FXZ2 was grown on PDA plates at 25°C for 7 days. Then, the mycelia were scraped off and suspended in autoclaved distilled water and divided equally into two portions (A and B). Suspension B was autoclaved at 121°C for 20 min. The pots were randomly divided into two groups (I and II). Further, the plants of group-I were sprayed with suspension A (E+) and group II with autoclaved suspension B (E-) at different time intervals 7, 15, 30, and 45 days of the transplant. The plants were harvested after growing for 60 days, and the fresh leaves were collected from E+ and E- and flash frozen right away with liquid nitrogen, preserved at −80°C, and used within 2 weeks for biochemical analysis. Simultaneously, the rhizospheric soil from each pot was collected, air-dried, and kept in poly-bags with proper labels for subsequent analysis.

### Plant growth parameters

#### Shoot, root length, and the dry biomass

The harvested plants were washed under tap water and finally rinsed with deionized water. After that, the plants were divided into shoots (all aboveground parts) and roots (all belowground parts), and the length was measured. Finally, the shoots and roots were oven-dried at 50–60°C to constant weight, and then the dry biomass was recorded. The dried plant samples were used for metal content analysis.

#### Total chlorophyll content

Ten plants were selected randomly from each group before harvesting, and the total chlorophyll content of the youngest fully developed leaves of each plant was analyzed using a chlorophyll meter (SPAD-502Plus, Konica Minolta, Inc., Tokyo, Japan). And the final chlorophyll content of each group was an average of 10 plants.

#### Lipid peroxidation

A chemical assay kit (Nanjing Jiancheng Bioengineering Institute, Nanjing, China) was used to measure the lipid peroxidation extent, which was expressed in nanomoles of malondialdehyde (MDA) formation per gram of tissue. Three replicates were made. To do this, the frozen leaves’ tissue was crushed in a chilled phosphate buffer (50 mM, pH 7.2). Then, the homogenate was centrifuged for 10 min at 3,500 rpm and 4°C. After that, the supernatant was transferred to a new tube and the MDA was measured spectrophotometrically (MAPADA UV-1800 PC).

#### Glutathione content

The total glutathione (T-GSH) and oxidized glutathione (GSSG) assay kits were used for GSH analysis (Nanjing Jiancheng Bioengineering Institute, Nanjing, China). To do this, the frozen leaves were homogenized in an extraction buffer (1:4 ratio, wt/vol). Then, the homogenate was centrifuged for 10 min at 3,500 rpm and 4°C. After that, the supernatant was used for GSH analysis (Rahman et al., 2006).

The absorbance of the assay mixture was measured according to the manufacturer’s protocol, and the T-GSH and GSSG content was calculated using the given formulas. The GSH content was expressed in micromoles per gram of fresh leaves, which was the calculated difference of GSSG content from the T-GSH content according to the formula mentioned in the kit.

### Cd/Zn accumulation in the plants

The dried root/shoot samples were homogenized into fine powders, respectively. Then, 0.2 g powders were digested in 5 ml HNO_3_ (65% w/w) at 110°C for 2 h. After cooling 1 ml H_2_O_2_ (30% w/w) was added and the mixture was heated for 1 h. The digests were then diluted to 50 ml with triple-distilled water (Shen et al., 2013). Finally, the concentrations of Cd/Zn were estimated by flame atomic absorption spectrometry (Li et al., 2014). The test was performed in triplicate.

### Chemical speciation of Cd/Zn in rhizospheric soils

The chemical speciation of Zn/Cd in rhizospheric soils was tested according to the method of Tessier et al. (1979). The method consists of five steps that give rise to five fractions operationally defined as F1 (exchangeable), F2 (carbonate bound), F3 (Fe-Mn oxides bound), F4 (organic bound), and F5 (residual). Briefly, 1 gm fine powder of the soil was taken into a 50-ml polycarbonate centrifuge tube. First fraction was extracted with 20 ml 1.0 M MgCl_2_ (pH 7.0) for 1 h with continuous agitation. The second fraction was extracted with 10 ml 1.0 M sodium acetate (pH adjusted to 5.0 with acetic acid) for 5 h with continuous agitation. The third fraction was extracted with 20 ml 0.04 M NH_2_OH.HCl in 25% sodium acetate (pH 2.0) for 6 h at 96°C in a water bath with occasional agitation. The fourth fraction was extracted with 3 ml 0.02 M HNO_3_ and 5 ml 30% H_2_O_2_ (pH adjusted to 2.0 with HNO_3_) for 2 h at 96°C in a water bath with occasional agitation; after that, 3 ml 30% H_2_O_2_ (pH 2.0 with HNO_3_) was added and extracted for 2 h at 96°C in a water bath with occasional agitation; subsequently, after cooling, 5 ml 3.2 M ammonium acetate in 20% (v/v) HNO_3_ was added, and the samples were diluted to 20 ml and agitated continuously for 30 min. The fifth fraction was the residue left from the organic fraction. It was digested with 4 ml HCl-HNO_3_ (3:1, v/v) mixture at 80°C for 30 min, then 100°C for 30 min, and finally 120°C for 1 h. After that, cooled and 1 ml HClO_4_ was added to continue digestion at 100°C for 20 min, followed by 120°C for 1 h. The concentrations of Zn/Cd were determined by flame atomic absorption spectrometry in different fractions (Li et al., 2014). Triplicates were made. The effect of FXZ2 inoculation (FE) was introduced to evaluate the influence on the chemical speciation of Zn/Cd in the rhizospheric zone. Here, FE = (F*^E+^* - F*^E–^*)/F*^E–^*, where F*^E+^* and F*^E–^* represent the corresponding fractions of metals in the E+ and E- treatments, respectively. The FE data were represented as heatmap drawn using Heatmap function of R version 4.1.1 (2021).

## Statistical analysis

Boxplots were drawn using the ggboxplot function of the ggpubr package (version “0.4.0.999”) in R version 4.1.1 (Core TeamR, 2021) and RStudio 2021.09.0 (R Studio Team, 2021). The difference between E+ and E- was determined using Student’s *t*-test significant at the level of <0.05% performed in RStudio and one-way ANOVA and Duncan test (*p* < 0.05).

## Results and discussion

### The effect of FXZ2 on *Dysphania ambrosioides* growth

**FIGURE 1:**
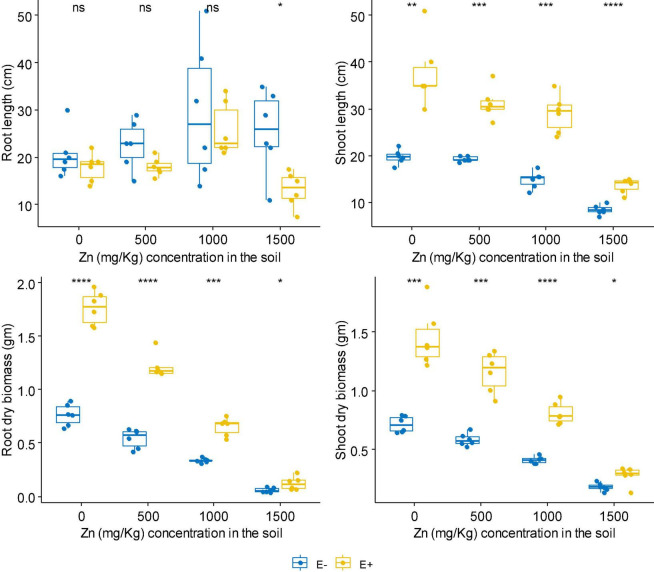
The effect of FXZ2 on the growth of *Dysphania ambrosioides* under Zn stress (*p < 0.05, **p < 0.005, ***p < 0.0005, ****p < 0.00005, ns *p* > 0.05 *t*-test).

**FIGURE 2:**
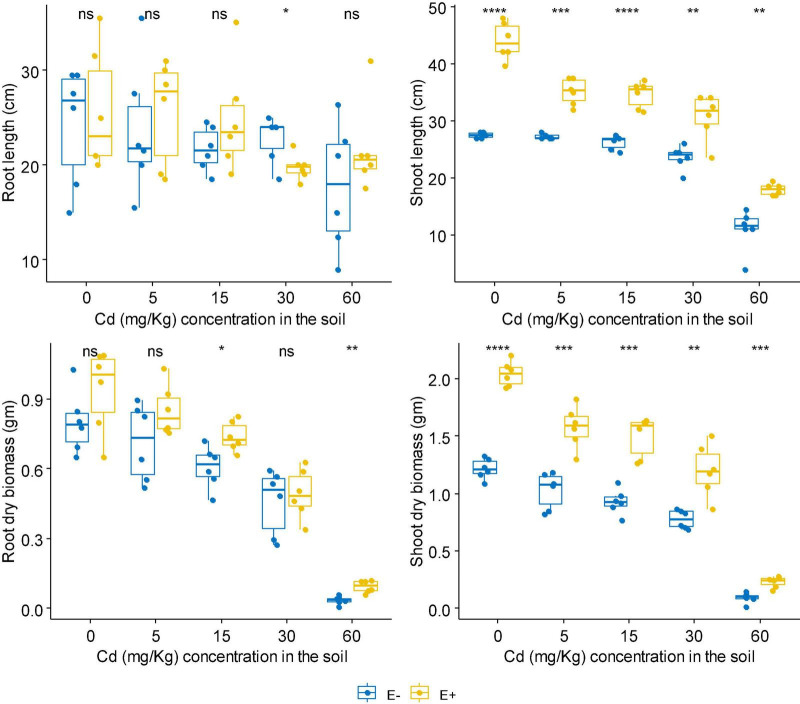
The effect of FXZ2 on the growth of *Dysphania ambrosioides* under Cd stress (*p < 0.05, **p < 0.005, ***p < 0.0005, ****p < 0.00005, ns *p* > 0.05 *t*-test).

No matter at Zn or Cd stress, FXZ2 significantly improved the shoot length of *D. ambrosioides* (*p* < 0.05) (Figures 1, 2). However, it had different effects on the root length and dry biomass of *D. ambrosioides* under Zn stress and Cd stress. At all Zn concentrations, FXZ2 decreased the root length of *D. ambrosioides*, but the difference was only significant (*p* < 0.05) at 1,500 mg kg^–1^ Zn stress (Figure 1). Both the dry biomass of shoots and roots of E+ were significantly (*p* < 0.05) higher than those of E- at all Zn concentrations. Contrary to this, at all Cd concentrations, FXZ2 improved the root length of *D. ambrosioides* (*p* > 0.05) except at 30 mg kg^–1^ Cd stress (*p* < 0.05) (Figure 2). The dry biomass of E+ shoots was significantly (*p* < 0.05) higher than that of E- shoots. However, the dry biomass of E+ roots was more than that of E- roots at all Cd concentrations, but the difference was only significant (*p* < 0.05) at 15 and 60 mg kg^–1^ Cd stress.

Although Zn is an essential element required for plant growth, its high concentration in the soil could affect essential plant metabolic functions and cause retarded growth and senescence (Yadav, 2010). High Cd concentration negatively affects mineral nutrition and carbohydrate metabolism and consequently decreases plant biomass production (John et al., 2009). Increased Cd also alters the activity of antioxidant enzymes, including superoxide dismutase, peroxidase, etc. (Sun et al., 2007). In the present study, it was found that with the increase of Zn/Cd concentration in the soil, both the dry biomass of E+ and E- shoots and roots decreased (Figures 1, 2). But still, the dry biomass of E+ was better than E-. The finding suggests that fungal seed endophyte FXZ2 improved *D. ambrosioides* growth under different Zn/Cd stress. These results are similar to previous studies that microbial inoculation positively affected the plant biomass under Zn and/or Cd stress (He et al., 2013; Bilal et al., 2018; Singh et al., 2018; Zhu et al., 2018; Zhai et al., 2022). In addition, the present study showed that the plant exposure to Cd stress affects the biomass in a dose-dependent manner; similar observations were also reported by other authors (Sun et al., 2007; Kamran et al., 2015; Khan et al., 2015; Shahid et al., 2019; Zhang et al., 2019).

In general, FXZ2 induced enhancement of plant growth indicators such as shoot and root lengths. Their dry weight indicates a plant’s ability to tolerate Zn and Cd stress and has shown positive growth (Kamran et al., 2015). Both bacterial and fungal endophytes have been linked to the improved plant growth-related characteristics of the host plants under metal stress (Bilal et al., 2018; Zhu et al., 2018; Shahid et al., 2019; Rattanapolsan et al., 2021; Hussain et al., 2022).

### The effect of FXZ2 on *Dysphania ambrosioides* Zn/Cd accumulation

The uptake and accumulation of Zn/Cd in the shoots and roots of E+ and E- are shown in Table 1. Generally, the Zn concentrations in E+ and E- plants differed from the Zn concentration in the soil (Table 1). At 0 mg kg^–1^ Zn stress, the Zn content in the shoots of E+ plants was significantly (*p* < 0.05) high than that of E- plants, however, this was only slightly more (*p* > 0.05) in the roots of E+ plants. Contrary to this, at 500 and 1,000 mg kg^–1^ Zn stress, the shoot Zn content in E+ plants was significantly (*p* < 0.05) lower than that in E- plants, while only slightly more (*p* > 0.05) in E+ plants at 1,500 mg kg^–1^ Zn treatment. Similarly, the root Zn content was more (*p* > 0.05) in the E- plants than E+ plants at 500 and 1,000 mg kg^–1^ Zn treatment, while less (*p* > 0.05) in E- plants at 0 and 1,500 mg kg^–1^ Zn treatments.

**TABLE 1:** Zn/Cd accumulation in the shoots and roots of FXZ2 inoculated plants (E+) and non-inoculated plants (E-).

The original concentration of Zn/Cd in the soils (mg kg^–1^)		The treatment of FXZ2	The concentration of Zn/Cd in the plants (mg kg^–1^)*	
			Shoots	Roots
Zn	0	E-	253.83 ± 4.83a	142.03 ± 19.15a
		E+	500.55 ± 69.23b	197.67 ± 110.19a
	500	E-	2,113.00 ± 113.86d	660.50 ± 65.30ab
		E+	1,850.00 ± 36.72c	591.20 ± 17.41ab
	1,000	E-	2,646.33 ± 79.10e	1,766.33 ± 453.99c
		E+	2,184.67 ± 190.17d	1,281.33 ± 90.98bc
	1,500	E-	3,180.33 ± 48.79f	3,074.33 ± 947.53d
		E+	3,370.33 ± 236.05f	3,576.33 ± 345.30d
Cd	0	E-	0.16 ± 0.08a	0.32 ± 0.11a
		E+	0.14 ± 0.02a	0.27 ± 0.13a
	5	E-	5.10 ± 0.07ab	23.75 ± 1.56ab
		E+	8.39 ± 1.30ab	24.91 ± 8.18ab
	15	E-	12.28 ± 0.98abc	43.23 ± 6.63ab
		E+	16.97 ± 0.88bc	59.50 ± 6.73ab
	30	E-	23.48 ± 0.75cd	97.28 ± 24.17bc
		E+	34.48 ± 3.98d	167.08 ± 24.79c
	60	E-	91.09 ± 14.61e	445.72 ± 125.30d
		E+	120.79 ± 16.51f	739.48 ± 38.62e

*The values are Mean ± Std, *n* = 3; The different letters indicate the significant difference (*p* < 0.05, one-way ANOVA, Duncan test) between the individual plant part and metal in the different treatments.

The results suggest that the effect of FXZ2 on Zn uptake and accumulation was variable with the Zn content in the soil. Bilal et al. (2018) reported that the consortia endophytic microbes decreased Al and Zn content in the shoots and roots of *Glycine max* L. under 2.5 mM Al and Zn stress. Garg and Singh (2018) found that *Rhizophagus irregularis* combined with silicon amended soil and individually also decreased leaves and roots Zn content under Zn stress (600 and 1,000 mg kg^–1^). While the other studies showed different results; for example, the endophytic bacterium *Sphingomonas* sp. increased Zn uptake in *Sedum alfredii* (Chen et al., 2014). Similarly, dark septate endophyte *Exophiala pisciphila* increased Pb, Zn, and Cd content in the roots and decreased in the shoots of *Zea mays* L. (Li et al., 2011); rhizobacterium *Enterobacter ludwigii* increased the Zn content in wheat under metal stress (Singh et al., 2018). This indicates that different microbes have different effects on host plant metal accumulation. Therefore, artificial manipulation of these microbes can be exploited to achieve the desired beneficial response.

At 0 mg kg^–1^ Cd stress, the Cd content was more (*p* > 0.05) in the shoots and roots of E- than E+ plants. However, the shoot and root Cd contents were higher in E+ plants at all Cd treatments than those in E- plants. The difference was significant (*p* < 0.05) at 60 mg kg^–1^ Cd stress, while the difference was non-significant (*p* > 0.05) at 5, 15, and 30 mg kg^–1^ Cd (Figures 1, 2 and Table 1). FXZ2-induced Cd content increase in the shoots and roots was consistent with other studies (Ren et al., 2006; Soleimani et al., 2010; Wan et al., 2012; Deng et al., 2013; He et al., 2013). Besides, plant growth-promoting bacteria such as *Rhizobium sullae* and *Pseudomonas* sp. (Chiboub et al., 2019), arbuscular mycorrhizal fungi (Berthelot et al., 2018; Rafique et al., 2019), and arbuscular mycorrhiza and silicon amended soil in combination as well as alone (Garg and Singh, 2018) were also found to increase Cd accumulation in host plants. However, the finding was opposite to some previous studies that reported relatively lower Cd content in the roots and shoots and roots of the endophyte inoculated plants under Cd stress (Wang et al., 2016; He et al., 2017; Zhan et al., 2017; Shahid et al., 2019). Nevertheless, it is interesting to note that in both cases, growth-promoting endophyte inoculation has potential applications: If the endophyte can increase metal accumulation in host plants, it can be potentially used in phytoextraction. On the other hand, if the endophyte can decrease metal accumulation in host plants, it can be potentially used to reduce the metal content of agriculturally important crops to safe levels of consumption. Generally, metal contents in plant samples depend on the bioavailability of metals in soil (Kim et al., 2015), but this study provides sufficient evidence that endophytes can affect metal accumulation and growth under metal stress (Figures 1, 2 and Table 1).

### The effect of FXZ2 on Zn/Cd speciation in rhizospheric soils

**FIGURE 3:**
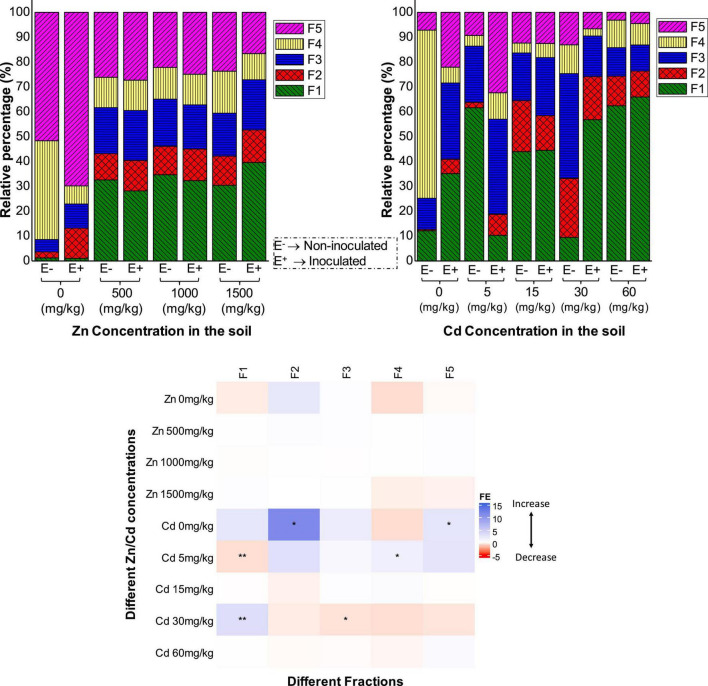
The effect of FXZ2 inoculation (FE) on the chemical forms of Zn/Cd in rhizospheric soils of *Dysphania ambrosioides* under Zn/Cd stress. F1: exchangeable fraction; F2: carbonate-bound fraction; F3: Fe-Mn oxides bound; F4: organic bound fraction; F5: residual fraction. The asterisks indicate a significant difference between F^E+^ and F^E–^ (*p < 0.05, **p < 0.005, *t*-test).

Zinc and Cd chemical speciation in rhizospheric soils of E+ and E- plants were shown in Figure 3. It was found that under Zn stress (500, 1,000, and 1,500 mg kg^–1^ Zn), most of Zn was in F1 (exchangeable fraction). Interestingly, at 500 and 1,000 mg kg^–1^ Zn stress, the Zn content of F1 + F2 was relatively less in rhizospheric soils of E+ than E- plants, while it was rather more in E+ plants in the 1,500 mg kg^–1^ Zn treatments. This can be correlated to the Zn concentration in the shoots and roots of E+ and E- plants in 500, 1,000, and 1,500 mg kg^–1^ Zn treatments. The metal in F4 (organic matter-bound fraction) and F5 (residual fraction) was the least available to plants. Together, these fractions were found relatively more in E+ than E- plants in 500 and 1,000 mg kg^–1^ Zn treatments, while it was relatively less in E+ plants in the 1,500 mg kg^–1^ Zn treatments. Results differed from previous studies, in which arbuscular mycorrhizal fungi (AMF) and plant growth-promoting rhizobacteria (PGPR) inoculation increased soil Zn mobility by changing Zn to high available fractions from low available fractions (Boostani et al., 2016).

Under Cd stress, no definite trend was observed in the relative percentage of the different fractions, especially at the low Cd stress (5 and 15 mg kg^–1^ Cd), while under high Cd exposure (30 and 60 mg kg^–1^ Cd), F1 + F2 were higher in E+ than E- plants. Wang et al. (2016) also reported a difference in the chemical speciation of Cd in the dark septate endophyte inoculated maize. In another study, endophyte inoculation to *Brassica juncea* increased F1 + F2 fractions of Cd in the rhizosphere compared to the control plants (Wang et al., 2020). The possible mechanism of the distinct shift in the chemical speciation of an element in rhizospheric soils is by modifying pH through the secreted root exudates (Long et al., 2013). Endophyte inoculation could affect the subcellular fractions of Cd in the host plant and its chemical forms. For example, AMF colonization increases Cd accumulation in *Medicago sativa* L. by changing Cd into inactive forms, having low toxicity (Wang et al., 2012). Similar AMF colonization affected Cd uptake and subcellular distribution by changing Cd chemical speciation in rice (Li H. et al., 2016; Luo et al., 2017). Besides, the observed results of Zn and Cd speciation might affect the anions and pH from ZnSO_4_.7H_2_O and CdCl_2_⋅2.5H_2_O supplemented to induce Zn and Cd stress, respectively (Wang et al., 2016).

FXZ2 inoculation affected the chemical speciation in root zone soils of *D. ambrosioides* only to some extent. The effect of FXZ2 inoculation (FE) was variable for the different fractions of Zn and Cd in rhizospheric soils (Figure 3). The effect was not significant for all fractions of Zn in the different treatments, while in the case of Cd, there were six significant alterations out of a total 25 alterations by FXZ2 inoculation. Chemical speciation in the rhizosphere regulates toxicokinetics, i.e., the uptake and translocation of metals by the plants from the root zone (Uchimiya et al., 2020). The manipulation of the phytomicrobiome can change the rhizosphere by the secretion of root exudates, which can alter the microbial signaling compounds and chemical speciation (Bhatt et al., 2020). It has to be noted that in this study, we evaluated the chemical speciation in the rhizosphere soil only at the time of harvest (60 days). It would be interesting to evaluate how the chemical speciation of metals changes in the rhizosphere when the plant is inoculated with FXZ2 during different time intervals as the plant grows in metal stress conditions and further how it affects the rhizosphere microbial community.

### The effect of FXZ2 on biochemical factors of *Dysphania ambrosioides*

**FIGURE 4:**
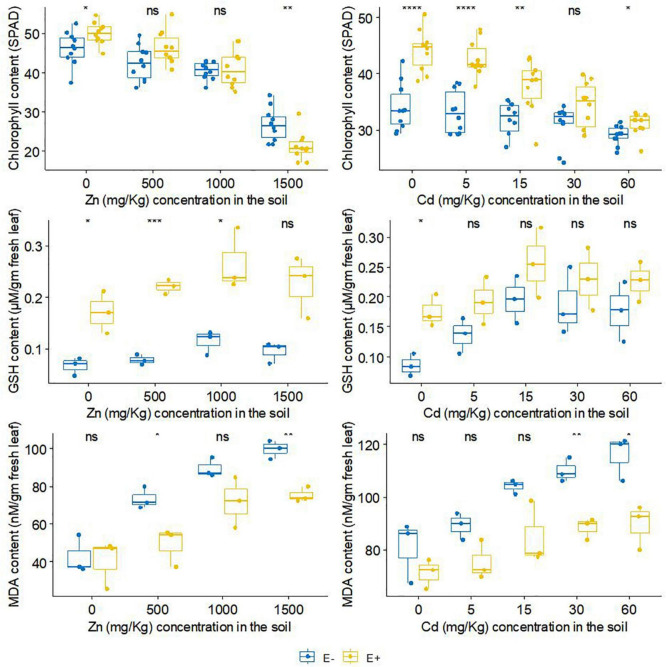
The effect of FXZ2 on the Chlorophyll, GSH and MDA content of *Dysphania ambrosioides* under Zn/Cd stress (*p < 0.05, **p < 0.005, ***p < 0.0005, ****p < 0.00005, ns *p* > 0.05 *t*-test).

FXZ2 inoculation had a positive effect on the total chlorophyll content of host plants (Figure 4). With the exception of 1,500 mg kg^–1^, E+ plants had a relatively higher total chlorophyll content in Zn treatments than E- plants. The differences were significant at 0 and 1,500 mg kg^–1^ Zn while non-significant (*p* > 0.05) at 1,000 mg kg^–1^ Zn. In Cd treatments, FXZ2 colonization significantly (*p* < 0.05) increased the total chlorophyll content of the host plants except at 30 mg kg^–1^ Cd stress (*p* > 0.05). With the increase of Zn and Cd concentration in the soil, the total chlorophyll content was decreased both for E+ and E- plants. The chlorophyll content is a significant indicator of plant growth status (Chen et al., 2010). Exceptionally high Zn in the soil can cause stress in plants, leaf chlorosis, and reduce photosynthesis (Broadley et al., 2007). Moreover, Cd-induced toxicity can adversely affect the plant chlorophyll biosynthesis by preventing δ-aminolevulinic acid dehydratase, porphobilinogen deaminase, and protochlorophyllide reductase activity and changing the photosynthetic electron transport at PS-II (Zulfiqar et al., 2021).

Our results supported the finding that the chlorophyll content decreased for the toxicity of Zn or Cd (Zhang et al., 2010; Kamran et al., 2015; Bilal et al., 2018). However, the chlorophyll of E+ plants was relatively higher than that of E- plants. The results agree with Hunt et al. (2005), who recorded that endophyte inoculation to perennial ryegrass increased chlorophyll content. Bilal et al. (2018) also reported that endophytic microbial consortia could significantly enhance the chlorophyll content of the inoculated plants under normal and Al/Zn stress. The low chlorophyll content under the influence of abiotic stress is generally due to the stress-related ROS generation and membrane lipid peroxidation, which further affects the fluidity and selectivity of the membrane (Verma and Mishra, 2005). Furthermore, in plant tissue metal stress results in the generation of ROS, which in the form of hydrogen peroxide and superoxide anion mimic and interrupt normal cellular functions by changing the oxidation/reduction cycle (Khan et al., 2015).

Tripeptide glutathione is one of the crucial plant metabolites having an essential role in the plant defense system as a ROS scavenging molecule. In plants, it occurs mainly in reduced form (GSH), and abundant production in the stress-adapted plant is related to a strongly activated defense system (Gill and Tuteja, 2010). The GSH analysis showed that FXZ2 inoculation affected the GSH content of host plants (Figure 4). In general, the GSH content of E+ plants was higher than E- plants under both Zn and Cd stress. The differences were significant (*p* < 0.05) at 500 and 1,000 mg kg^–1^ Zn stress, while under Cd stress, the difference was non-significant (*p* > 0.05). The thiol group of the glutathione is of high-affinity nature, linked to the complexation and detoxification of metals as a chelating compound, and takes part in the antioxidant process (Schat et al., 2002; Yadav, 2010; Cao et al., 2018). Further, it reduces phytotoxicity by forming an inactive glutathione-Cd complex and subcellular compartmentalization (Adamis et al., 2004; Zhang et al., 2019). The higher GSH content in E+ than E- plants suggests the inoculated endophyte induced counteractive mechanisms to check oxidative stress related to metal toxicity. Previous studies also indicated that inoculation of endophytic microbe can enhance the growth and tolerance of host plants to metal stress through GSH regulation, though the effect on GSH can vary with stress (Khan et al., 2015; He et al., 2017; Zhan et al., 2017).

Metal stress induces oxidative damage in plants, causing lipid peroxidation that disturbs cellular functions and membrane integrity; the injuries can be irreversible (Wan et al., 2012; Khan and Lee, 2013; Khan et al., 2015; Bilal et al., 2018). Malondialdehyde (MDA) is a byproduct of lipid peroxide breakdown. Lower MDA in plant tissue signifies lesser lipid peroxidation. The MDA content of different treatments is presented in Figure 4. It was found that FXZ2 inoculation lowered the MDA content of host plants. The differences were significant (*p* < 0.05) at 500 and 1,500 mg kg^–1^ Zn stress and higher Cd stress (30 and 60 mg kg^–1^). The relatively lower MDA in E+ plants suggests that the endophyte FXZ2 had a synergistic role against the oxidative stress due to elevated Zn and Cd. Results from this study are consistent with previous research that endophyte-infected plants had lower MDA contents, for instance, *Achnatherum inebrians* inoculated with endophyte *Neotyphodium gansuense*, and *Solanum nigrum* inoculated with endophyte *Serratia nematodiphila* under Cd stress (Zhang et al., 2010; Wan et al., 2012; Khan et al., 2015), *Glycine max* L. inoculated with endophytic fungus *Paecilomyces formosus* and bacteria *Sphingomonas* sp. under Al/Zn stress (Bilal et al., 2018), and tomato inoculated with two dark septate endophytes *Phialophora mustea* under Zn/Cd stress (Zhu et al., 2018).

### Phytohormone production by FXZ2

Phytohormone indole acetic acid (IAA) is responsible for apical dominance, cell elongation, evolution of vascular tissue, and improvement of plant stress tolerance (Wang et al., 2001; Eyidogan et al., 2012). And gibberellic acid (GA) is primarily responsible for seed germination, stem elongation, flower and trichome initiation, fruit development, and leaf expansion (Yamaguchi, 2008; Liu et al., 2009). Jasmonic acid (JA) has been demonstrated as a significant signaling molecule during plant defense, such as pathogens attack (Qi et al., 2016) and metals stress (Bilal et al., 2017; Per et al., 2018). JA was also reported to alter antioxidant potential, reduce H_2_O_2_ and MDA concentrations, and improve photosynthetic pigments concentrations under Pb and Cd stress in different plants (Piotrowska et al., 2009; Ahmad et al., 2017). Some endophytes can exogenously produce phytohormones to mitigate the effects of abiotic stress to host plants (Khan et al., 2012; Bilal et al., 2018; Chang et al., 2021). In the present study, it was found that FXZ2 exogenously secretes IAA (3.21 ± 0.59 μM L^–1^), GA (13.76 ± 0.20 pM L^–1^), and JA (257.70 ± 43.04 pM L^–1^) in liquid culture. These phytohormones may play some roles in plant growth and stress tolerance under Zn/Cd stress. Similarly, some phytohormones producing fungal species, e.g., *Fusarium oxysporum*, *Piriformospora indica*, *Phoma glomerata*, *Penicillium* sp., and *Exophiala pisciphila*, have found to improve host plants’ growth and crop productivity (Hasan, 2002; Yuan et al., 2010; Waqas et al., 2012; He et al., 2017). Further, the effect of FXZ2 on the endogenous production of phytohormones and host plants growth under metal stress can be tested on mutant plant cultivars not able to produce phytohormones, e.g., Waito-C (GA deficient mutant rice cultivar) (Khan et al., 2012). This can be a reliable future strategy to know how this endophyte improves the phytohormone content of the host plant and subsequently their growth under metal stress.

## Conclusion

Under variable Zn/Cd stress, seed endophyte FXZ2 significantly improved *D. ambrosioides* growth and its chlorophyll and GSH content. Our results demonstrated that FXZ2 inoculation transformed the Zn/Cd speciation in the rhizosphere of host plants, subsequently affecting their uptake and accumulation. The readily available fractions, i.e., exchangeable and carbonate-bound (F1 + F2) fractions of Zn decreased in E+ as compared to E- plants at 500 and 1,000 mg kg^–1^ Zn stress, congruently, Zn in shoots of E+ plants decreased significantly (*p* < 0.05). However, under Cd stress (30 and 60 mg kg^–1^), the effect was different, the Cd concentration in F1 + F2 increased in rhizospheric soils of E+ plants, and subsequently, Cd accumulation in E+ plants was significantly (*p* < 0.05) increased. Therefore, FXZ2 can have different applications, for example, in agriculturally important crops it can be used to improve Zn tolerance in contaminated soils or in phytoextraction by increasing Cd bioaccumulation at high Cd stress.

Moreover, FXZ2 could exogenously secrete phytohormones IAA, GA, and JA, which could be a key mechanism for promoting host plants’ growth under Zn/Cd stress. Further study is required to investigate the role of FXZ2 in the endogenous production of phytohormones in inoculated plants.

## Data availability statement

The original contributions presented in this study are included in the article/supplementary material, further inquiries can be directed to the corresponding author.

## Author contributions

VS: conceptualization, methodology, writing—original draft, investigation, formal analysis, and data curation. SP: conceptualization, methodology, writing—original draft, investigation, formal analysis, and data curation. WT: project investigation and resources and project administration. HH: writing—review and editing and validation. JW: writing—review and editing. HL: conceptualization, supervision, writing—review and editing, validation, resources, funding acquisition, and project administration. All authors contributed to the article and approved the submitted version.

## Footnotes

1
http://www.ncbi.nlm.nih.gov/
